# [^111^In]In/[^177^Lu]Lu-AAZTA^5^-LM4 SST_2_R-Antagonists in Cancer Theranostics: From Preclinical Testing to First Patient Results

**DOI:** 10.3390/pharmaceutics15030776

**Published:** 2023-02-26

**Authors:** Berthold A. Nock, Panagiotis Kanellopoulos, Euy Sung Moon, Maritina Rouchota, George Loudos, Sanjana Ballal, Madhav P. Yadav, Chandrasekhar Bal, Prashant Mishra, Parvind Sheokand, Frank Roesch, Theodosia Maina

**Affiliations:** 1 Molecular Radiopharmacy, INRaSTES, NCSR “Demokritos”, 15310 Athens, Greece; 2 Department Chemie, Standort TRIGA, Johannes Gutenberg-Universität Mainz, D-55126 Mainz, Germany; 3 BIOEMTECH, Lefkippos Attica Technology Park, NCSR “Demokritos”, 15310 Athens, Greece; 4 Department of Nuclear Medicine, AIIMS, Ansari Nagar, New Delhi 110029, India

**Keywords:** somatostatin subtype 2 receptor (SST_2_R)-antagonist, AAZTA^5^ chelator, AAZTA^5^-LM4, theranostics, neuroendocrine tumors, In-111, Lu-177, SPECT imaging, radionuclide therapy

## Abstract

Aiming to expand the application of the SST_2_R-antagonist LM4 (DPhe-c[DCys-4Pal-DAph(Cbm)-Lys-Thr-Cys]-DTyr-NH_2_) beyond [^68^Ga]Ga-DATA^5m^-LM4 PET/CT (DATA^5m^, (6-pentanoic acid)-6-(amino)methy-1,4-diazepinetriacetate), we now introduce AAZTA^5^-LM4 (AAZTA^5^, 1,4-bis(carboxymethyl)-6-[bis(carboxymethyl)]amino-6-[pentanoic-acid]perhydro-1,4-diazepine), allowing for the convenient coordination of trivalent radiometals of clinical interest, such as In-111 (for SPECT/CT) or Lu-177 (for radionuclide therapy). After labeling, the preclinical profiles of [^111^In]In-AAZTA^5^-LM4 and [^177^Lu]Lu-AAZTA^5^-LM4 were compared in HEK293-SST_2_R cells and double HEK293-SST_2_R/wtHEK293 tumor-bearing mice using [^111^In]In-DOTA-LM3 and [^177^Lu]Lu-DOTA-LM3 as references. The biodistribution of [^177^Lu]Lu-AAZTA^5^-LM4 was additionally studied for the first time in a NET patient. Both [^111^In]In-AAZTA^5^-LM4 and [^177^Lu]Lu-AAZTA^5^-LM4 displayed high and selective targeting of the HEK293-SST_2_R tumors in mice and fast background clearance via the kidneys and the urinary system. This pattern was reproduced for [^177^Lu]Lu-AAZTA^5^-LM4 in the patient according to SPECT/CT results in a monitoring time span of 4–72 h pi. In view of the above, we may conclude that [^177^Lu]Lu-AAZTA^5^-LM4 shows promise as a therapeutic radiopharmaceutical candidate for SST_2_R-expressing human NETs, based on previous [^68^Ga]Ga-DATA^5m^-LM4 PET/CT, but further studies are needed to fully assess its clinical value. Furthermore, [^111^In]In-AAZTA^5^-LM4 SPECT/CT may represent a legitimate alternative diagnostic option in cases where PET/CT is not available.

## 1. Introduction

The successful advent of theranostic octreotide analogs in the clinic to combat somatostatin subtype 2 receptor (SST_2_R)-expressing neuroendocrine tumors (NETs), such as the recently approved theranostic pair [^68^Ga]Ga/[^177^Lu]Lu-DOTA-TATE, has paved the way for new exciting developments in the field of receptor-targeted diagnostic imaging and radionuclide therapy of human tumors [1,2,3,4,5,6,7]. In this concept, radionuclide therapy is based on previous PET/CT to identify patients eligible to receive it, perform dosimetry and optimize therapeutic schemes. Furthermore, post-therapy PET/CT is applied to assess therapeutic responses and monitor disease progress in an approach abiding to modern personalized medicine principles [8,9]. A recent breakthrough in the field of NET theranostics has been the shift of paradigm from radiolabeled SST_2_R-agonists to antagonists [10,11,12,13,14,15,16,17]. Despite their inability to internalize in cancer cells, radiolabeled SST_2_R-antagonists were shown to bind to more receptor sites on cancer cells, comprising both active and non-active receptor populations, resulting in higher tumor uptake in animal models and patients. Moreover, background clearance turned out to be favorably faster for antagonists for reasons not yet fully understood. Hence, radiolabeled SST_2_R-antagonists are expected to dynamically enter the clinic in the future with the development of new radiopharmaceuticals of higher diagnostic contrast and therapeutic index and potentially also broader clinical indications.

Most hitherto-developed SST_2_R-directed radioligands, both agonists and antagonists, have been modified with the universal chelator DOTA (1,4,7,10-tetraazacyclododecane-N,N′,N″,N‴-tetraacetic acid), thereby allowing for stable coordination of a wide range of trivalent metals of medical interest [16,18,19]. However, labeling of DOTA-modified ligands with Ga-68, In-111 and Lu-177 requires prolonged heating at elevated temperatures [20]. This procedure may affect the chemical integrity of thermosensitive vectors and is logistically challenging in clinical practice, especially for the shorter-lived Ga-68 (t_1/2_ = 68 min) [21,22]. An alternative route to overcome this problem is the introduction of hybrid chelators which allow fast coordination of the radiometal at lower temperatures without compromising the stability of the forming radiometal chelate [23,24]. Thus, the bifunctional hybrid chelator DATA^5m^ ((6-pentanoic acid)-6-(amino)methy-1,4-diazepinetriacetate) has been coupled to a variety of interesting vectors, including SST_2_R-targeting peptide motifs, enabling the facile and stable coordination of radiogallium (Ga-67/68) [25,26,27,28,29]. Preclinical studies have acquired convincing evidence for the successful clinical application of resulting [^68^Ga]Ga-radioligands with PET/CT. We have been actively engaged in this effort by studying [^67/68^Ga]Ga-labeled TOC ([Tyr^3^]octreotide, SST_2_R-agonist) [26,27,28] and the new SST_2_R-antagonist LM4 (p-Cl-Phe-c[DCys-4Pal-DAph(Cbm)-Lys-Thr-Cys]-DTyr-NH_2_; 4Pal: (4-pyridyl)Ala, DAph(Cbm): D-4-(carbamoyl)amino-phenylalanine), derivatized by coupling DATA^5m^ at the N-terminus, in animal models and in patients [30]. The ease of labeling with radiogallium was confirmed, along with the exceptional performance of the new tracers in comparison with suitable DOTA-modified references. Furthermore, we could establish that [^67/68^Ga]Ga-DATA^5m^-LM4 displayed better biodistribution profile than [^67/68^Ga]Ga-DATA^5m^-TOC. 

In the present work, we were interested in expanding the clinical application prospects of LM4 beyond [^68^Ga]Ga-DATA^5m^-LM4 PET/CT, particularly focusing on radionuclide therapy options. Hence, our attention has been directed to alternative hybrid bifunctional chelators known to easily and stably coordinate Lu-177 under milder conditions than those commonly used for DOTA-derivatized vectors. Amongst various candidates, AAZTA (1,4-bis(carboxymethyl)-6-[bis(carboxymethyl)]amino-6-methylperhydro-1,4-diazepine) has triggered our interest. AAZTA was first proposed as a new structural entry for the development of improved Gd(III)-based magnetic resonance imaging (MRI) agents [31], but considered suitable as well for the coordination of Sc-44, Ga-68,Cu-64/67, Lu-177 and other medically relevant radiolanthanides [32,33,34,35,36,37,38,39,40,41]. A few years later, a bifunctional derivative of AAZTA with an additional carboxylic acid group attached via a four methylene chain was developed, known as AAZTA^5^ (1,4-bis(carboxymethyl)-6-[bis(carboxymethyl)]amino-6-[pentanoic-acid]perhydro-1,4-diazepine) [34,35,39,40]. This new bifunctional chelator and its variations have been coupled to a variety of vectors which were successfully labeled with theranostic radiometals, including Lu-177, and showed exceptional biological profiles in preclinical cell and animal models.

**Figure 1:**
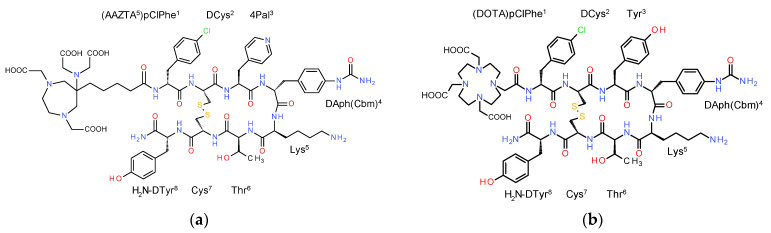
Chemical structures of (**a**) AAZTA^5^-LM4; (**b**) DOTA-LM3; the two analogs differ in the chelator (AAZTA^5^: 1,4-bis(carboxymethyl)-6-[bis (carboxymethyl)]amino-6-[pentanoic-acid]perhydro-1,4-diazepine and DOTA: 1,4,7,10-tetraazacyclododecane-1,4,7,10-tetraacetic acid) and in the residue at position 3: 4Pal (4-pyridyl)alanine) in LM4 and Tyr in LM3; DAph(Cbm): D-4-(carbamoyl)amino-phenylalanine).

In view of the above, we herein introduce AAZTA^5^-LM4 (Figure 1) for labeling with In-111 (for SPECT/CT) or Lu-177 (for radionuclide therapy) under lower-than-usual temperatures. The suitability of [^111^In]In/[^177^Lu]Lu-AAZTA^5^-LM4 for application in the management of SST_2_R-positive NETs was directly compared in HEK293-SST_2_R and wild-type (wt) HEK293 cells and tumor models with the respective [^111^In]In/[^177^Lu]Lu-DOTA-LM3 reference (LM3, p-Cl-Phe-c[DCys-Tyr-DAph(Cbm)-Lys-Thr-Cys]-DTyr-NH_2_), which is already applied in the clinic [42]. Eventually, the tissue distribution pattern of [^177^Lu]Lu-AAZTA^5^-LM4 was evaluated for the first time in a NET patient, based on previous [^68^Ga]Ga-DATA^5m^-LM4 PET/CT outcomes.

## 2. Materials and Methods 

### 2.1. Radiochemistry

#### 2.1.1. Chemicals, Ligands and Radionuclides

Common solvents and other chemicals were of reagent grade, but solvents for high-performance liquid chromatography (HPLC) were of HPLC grade. DOTA-LM3 (LM3: p-Cl-Phe-c[DCys-Tyr-DAph(Cbm)-Lys-Thr-Cys]-DTyr-NH_2_; DOTA: 1,4,7,10-tetraazacyclododecane-1,4,7,10-tetraacetic acid; DAph(Cbm): D-4-(carbamoyl)amino-phenylalanine) was obtained from PiChem Forschungs- und Entwickungs GmbH (Raaba-Grambach, Austria). AAZTA^5^-LM4 (LM4: p-Cl-Phe-c[DCys-4Pal-DAph(Cbm)-Lys-Thr-Cys]-DTyr-NH_2_; 4Pal: (4-pyridyl)Ala and AAZTA^5^: 1,4-bis(carboxymethyl)-6-[bis (carboxymethyl)]amino-6-[pentanoic-acid]perhydro-1,4-diazepine) was obtained from Peptide Specialty Laboratories GmbH (Heidelberg Germany). Analytical data, including purity determined by HPLC analysis and matrix-assisted laser desorption/ionization–time of flight (MALDI-TOF) mass spectrometry (MS) results, are compiled in Table S1 in the Supplementary Materials. TATE was acquired as previously described [43]. LTT-SS28 ([Leu^8^,DTrp^22^,Tyr^25^]SS28) was purchased from Bachem AG (Bubbendorf, Switzerland). The [^nat^In]In(NO_3_)_3_ and [^nat^Lu]Lu(NO_3_)_3_ salts were purchased from Sigma-Aldrich Inc. (St. Louis, MO, USA).

For labeling of analogs used in preclinical studies, In-111 in the form of [^111^In]InCl_3_ (400–600 MBq/mL in 0.05 mM HCl) was purchased from Curium Pharma (Petten, The Netherlands) and Lu-177 in the form of [^177^Lu]LuCl_3_ (3.7 GBq/mL in 0.04 MHCl, RLu-3, As >370 GBq/mg Lu) was obtained from POLATOM (Otwok, Poland). For the preparation of [^125^I-Tyr^25^]LTT-SS28 [44], [^125^I]NaI was provided by PerkinElmer (Waltham, MA, USA) in dilute sodium hydroxide solution (pH 8–11). Aliquots of the radioligand stock solution in 0.1% BSA-PBS buffer were kept at −20 °C and were used in competition binding experiments (molar activity of 74 GBq/μmol).

#### 2.1.2. Radiolabeling

The peptide conjugates used in the preclinical study were dissolved at 2 mg/mL in double-distilled H_2_O and were placed in 50 μL aliquots in separate Eppendorf Protein LoBind tubes which were subsequently stored at −20 °C. Labeling of peptide conjugates for preclinical tests was conducted as detailed below: 

*Labeling with In-111*. The following items were successively pipetted into an Eppendorf Protein LoBind^®^ centrifuge tube (capacity: 1.5 mL): (a) EtOH (100 µL), (b) [^111^In]InCl_3_ (150 μL, 370–740 MBq/mL in 0.05 M HCl), (c) a freshly prepared Na-PABA solution (35 µL, 0.5 M; Na-PABA: sodium *p*-aminobenzoate) and (d) the corresponding bioconjugate stock solution (15 µL, 30 µg, ≈15 nmol). The labeling reaction mixtures were incubated at 50 °C for 10 min in the case of AAZTA^5^-LM4 and at 80 °C for 30 min in the case of DOTA-LM3. Quality control was performed after DTPA was added to a final concentration of 0.1 mM as a scavenger of unbound In-111. All radioligands were obtained in radiochemical purities exceeding 98% at apparent molar activities of 3.7–7.4 MBq [^111^In]In/nmol conjugate. 

*Labeling with Lu-177*. Likewise, the following reagent solutions were pipetted into an Eppendorf Protein LoBind^®^ centrifuge tube (capacity: 1.5 mL): (a) EtOH (120 µL), (b) [^177^Lu]LuCl_3_ (150 µL, 740 MBq/mL in 0.04 M HCl), (c) the bioconjugate stock solution (20 µL, 40 µg, ≈20 nmol) and (d) a freshly prepared Na-PABA solution (25 µL, 0.5 M). The labeling reaction mixture of AAZTA^5^-LM4 was incubated at 50 °C for 30 min and the respective DOTA-LM3 mixture at 80 °C for 30 min. DTPA was added to the vials at a final concentration of 0.1 mM to scavenge traces of unbound Lu-177 before performing the quality control. Both [^177^Lu]Lu-radioligands were obtained in high radiochemical purities (>98%) at apparent molar activities of approximately 37 MBq [^177^Lu]Lu/nmol ligand.

#### 2.1.3. Quality Control of Radiolabeled Products

*For Preclinical Studies.* Reverse-phase high-performance liquid chromatography (RP-HPLC) was the used for the quality control. A Waters Chromatograph based on a 600E multi-solvent delivery system and coupled to parallel photometric (Waters 2998 photodiode array detector; Vienna, Austria) and radiometric (Gabi gamma-detector from Raytest, RSM Analytische Instrumente GmbH; Straubenhardt, Germany) detection modes was used for analyses. The Empower Software (Waters, Milford, MA, USA) was used to control the system. For the analysis, aliquots of the radiolabeling solution were loaded on a Symmetry Shield RP18 cartridge column (5 μm, 3.9 mm × 20 mm, Waters, Eschborn, Germany), which was eluted with the following linear gradient: 100%A/0%B to 40%A/60%B in 20 min, whereby A = 0.01% TFA in H_2_O (*v*/*v*) and B = MeCN (system 1). The radiochemical purity of all radioligands was >98%. Hence, radioligand solutions were applied without further purification in biological assays that followed. The quality of radioligand solutions was tested before and after the conclusion of experiments.

Alternative methods for the preparation and the quality control of [^177^Lu]Lu-AAZTA^5^-LM4, involving manual or module semi-automatic production modes as well as a radiochemical stability study, are included in the Supplementary Files. Moreover, descriptions of the preparation and quality control of [^68^Ga]Ga-DATA^5m^-LM4 and [^177^Lu]Lu-AAZTA^5^-LM4 administered to the patient are also reported therein.

#### 2.1.4. Preparation of [^nat^In]In-AAZTA^5^-LM4 and [^nat^Lu]Lu-AAZTA^5^-LM4

A stock solution (60 µL, 2 mM, 120 nmol) of the respective nitrate salt dissolved in 1 M sodium acetate buffer of pH 4.6 (for indium) and pH 5.0 (for lutetium) was added into an Eppendorf Protein LoBind^®^ centrifuge tube containing AAZTA^5^-LM4 precursor stock solution (60 µL, 120 µg, ≈60 nmol) and the mixture was heated at 75 °C for 1 h. Complete metal incorporation by AAZTA^5^-LM4 was revealed by HPLC adopting the PDA-UV detection mode. Analyte separations were achieved on a Waters XSelect CSH^TM^ C18 reverse phase column (5 μm, 4.6 mm × 150 mm) applying the following linear gradient system at a 1.0 mL/min flow rate: starting from 10% B to 15% B in 5 min, followed by a further 0.5%/min increase in B within the ensuing 60 min (system 2; A and B, as given above in 2.1.3.). Application of these conditions allowed for baseline separation of metal-tagged from metal-free AAZTA^5^-LM4, as reported below. Retention times (UV trace, *t*_R_ in min): AAZTA^5^-LM4, *t*_R_ = at 20.6 min; [^nat^In]In-AAZTA^5^-LM4, *t*_R_ = 19.6 min; [^nat^Lu]Lu-AAZTA^5^-LM4, *t*_R_ = 19.4 min. 

Only authorized personnel handled any solution containing beta-/gamma-emitting radionuclides in licensed facilities in compliance with European radiation safety guidelines. Protocols and facilities were supervised by the Greek Atomic Energy Commission (GAEC, license #A/435/17092/2019 and #A/435/15767/2019).

### 2.2. Cell Studies

#### 2.2.1. Cell Culture

Two cell lines were employed in the present study. Firstly, the wild-type (wt) HEK293 cells, lacking any measurable SST_2_R expression, served as negative controls. Then, HEK293 cells transfected to stably express the human SST_2_R tagged with the T7-epitope (HEK293-SST_2_R) were used, which were a kind gift of Prof. S. Schultz (Jena, Germany). Dulbecco’s Modified Eagle Medium (DMEM), containing Glutamax-I and supplemented with 10% (*v*/*v*) heat-inactivated fetal bovine serum (FBS), 100 U/mL penicillin and 100 μg/mL streptomycin, was used as cell culture medium. Cells were kept in a controlled humidified atmosphere with 5% CO_2_ at 37 °C. For transfected HEK293-SST_2_R cells, 400 μg/mL G418 was additionally introduced in the medium. Culture media were obtained from by Gibco BRL, Life Technologies (Grand Island, NY, USA) and supplements were purchased from Biochrom KG Seromed (Berlin, Germany). Cell passages were conducted at 70–85% confluency using a solution of trypsin/EDTA (0.05%/0.02% *w*/*v*).

#### 2.2.2. Competition Binding Experiments

Competition binding assays were conducted for AAZTA^5^-LM4, [^nat^In]In-AAZTA^5^-LM4 and [^nat^Lu]Lu-AAZTA^5^-LM4 against [^125^I-Tyr^25^]LTT-SS28 in freshly harvested HEK293-SST_2_R cell membranes. In brief, to assay tubes test compound (30 μL solution of increasing 10^−13^–10^−5^ M concentrations), radioligand (70 μL, 50 pM corresponding to ≈40,000 cpm) and membrane homogenate (200 μL) were added to a final volume of 300 μL in binding buffer (50 mM HEPES pH 7.4, 1% BSA, 5.5 mM MgCl_2_, 35 μM bacitracin). Samples in triplicate for each concentration point were incubated for 1 h at 22 °C in an Incubator-Orbital Shaker unit, (MPM Instr. SrI). Competition was abruptly stopped by the addition of ice-cold washing buffer and fast filtration through glass fiber filters (Whatman GF/B, pre-soaked for 2 h in a 1% polyethyleneimine (PEI) aqueous solution). This operation was contacted semi-automatically on a Brandel Cell Harvester (Adi Hassel Ingenieur Büro, Munich, Germany). They were subsequently rinsed with ice-cold washing buffer (10 mM HEPES pH 7.4, 150 mM NaCl). Filters were collected in plastic tubes and their radioactivity content was measured in an automated multi-sample well-type γ-counter (NaI(Tl) 3″ crystal-equipped Canberra Packard Cobra^TM^ Quantum U5003/1, Auto-Gamma^®^ instrument). Nonlinear regression for a one-site model was applied to determine the half-maximal inhibitory concentration (IC_50_) values using the PRISM^TM^ 6.0 GraphPad software (San Diego, CA, USA). Results from three independent experiments in triplicate were calculated and presented as mean IC_50_ ± standard deviation (sd) values.

#### 2.2.3. Radioligand Uptake in HEK293-SST_2_R Cells

The following radioligands were tested side by side: [^111^In]In-AAZTA^5^-LM4 together with [^111^In]In-DOTA-LM3 and [^177^Lu]Lu-AAZTA^5^-LM4 together with [^177^Lu]Lu-DOTA-LM3. A day before the experiment, HEK293-SST_2_R cells were measured and (1 × 10^6^/well) placed in poly-lysine-coated six-well plates in the incubator. The following day, the plates were placed on ice. They were then washed two times with ice-cold internalization medium (IM: DMEM Glutamax-I supplemented by 1% (*v*/*v*) FBS). The supernatant was removed by aspiration and the plates were left on the bench at ambient temperature. Fresh warmed medium was added (1.2 mL) to the plates, followed by a solution of test radioligand (50,000 cpm corresponding to 0.5 pmol total peptide in 150 μL 0.5% BSA PBS). Internalization medium was added in the upper three wells of each plate (150 μL, total) and a solution of TATE to a final concentration of 1 μM (150 μL, non-specific) in the lower three ones. The plates were left in the Incubator at 37 °C for 60 min and the incubation was interrupted by placing the plates on ice and collecting the medium. The cells were then washed with ice-cold 0.5% BSA-PBS (1 mL) and the washings collected. After incubation of cells (2 × 5 min) at ambient temperature in acid wash buffer (50 mM glycine in 0.1 M NaCl, pH 2.8), supernatants were collected (membrane-bound fraction). Cells were washed and aspirated with 0.5% BSA-PBS, then lysed by the addition of 1 N NaOH. Mechanical detachment of lysates allowed full collection in the corresponding tubes (internalized fraction). Samples were measured for their radioactivity content in the γ-counter. Cell-associated/internalized radioactivity was calculated vs. total added activity for each well. The respective specific values were determined by subtracting values in the presence of excess TATE (lower well triplicates—non-specific) from those without the addition of blocker (upper well triplicates—totals). Results from at least three independent experiments in triplicate were given as average ± sd values.

### 2.3. Animal Studies

#### 2.3.1. Stability Studies

For the assessment of metabolic stability of [^111^In]In-AAZTA^5^-LM4 and [^177^Lu]Lu-AAZTA^5^-LM4 in vivo, 6 healthy male Swiss albino mice were used (NCSR “Demokritos” Animal House, Athens, Greece; body weight: 30 ± 5 g). The animals received a 100 μL bolus via the tail vein containing either [^111^In]In-AAZTA^5^-LM4 or [^177^Lu]Lu-AAZTA^5^-LM4 (2.5–3 nmol of total conjugate in vehicle: saline/EtOH 9/1 *v*/*v*; these solutions corresponded to up to 11 MBq for In-111 and up to 74 MBq for Lu-177). Mice were euthanized 5 min post-injection (pi) and blood was rapidly retrieved from the heart with a pre-chilled 1 mL penicillin syringe. Blood samples were immediately placed in pre-chilled Eppendorf Protein LoBind^®^ tubes on ice, containing EDTA (40 µL, 50 mM Na_2_EDTA solution). Plasma was collected after centrifugation (10 min, 2000× *g*/4 °C, in a Hettich Universal 320R centrifuge, Tuttlingen, Germany), and an equal volume of cold MeCN was added. After a second centrifugation (10 min, 15,000× *g*/4 °C), supernatants were collected and concentrated to a small volume (≈50–100 μL) under a gentle N_2_ flux at 40 °C. Physiological saline (400 μL) was added and the resulting solution samples were passed through a Millex GV filter (0.22 μm, 13 mm Ø, Millipore, Milford, MA, USA). Filtrate aliquots were analyzed by radio HPLC for the detection of forming radiometabolites (system 1). The elution time (*t*_R_) of intact [^111^In]In-AAZTA^5^-LM4 or [^177^Lu]Lu-AAZTA^5^-LM4 was determined by co-injection of blood samples processed as above with an aliquot of the labeling solution. Results were obtained from three mice per radioligand and are presented as average percentage of intact radiopeptide ± sd.

#### 2.3.2. Biodistribution in SCID Mice Bearing Twin HEK293-SST_2_R and wtHEK293 Tumors

Freshly harvested HEK293-SST_2_R cells (1.2 × 10^7^cells) and wtHEK293 cells (0.6 × 10^7^ cells) were suspended in physiological saline (150 μL) and inoculated in the right and left flanks, respectively, of 43 male SCID mice (23.1 ± 1.6 g body weight, six weeks of age on arrival day; NCSR “Demokritos” Animal House, Athens, Greece). Mice were kept under aseptic conditions for 12–15 days and until palpable tumors (300–600 mg) were grown at the inoculation sites. On the day of biodistribution, animals were intravenously (iv) injected in groups of four with 100 μL bolus (vehicle: saline/EtOH 9/1 *v*/*v*) containing 74–92 kBq of either [^111^In]In-AAZTA^5^-LM4 or [^111^In]In-DOTA-LM3 and corresponding to 20–25 pmol AAZTA^5^-LM4 or DOTA-LM3, respectively. Animals were euthanized at 4 and 24 h pi and immediately dissected. Blood samples, organs of interest and tumors were rapidly collected, weighed and counted in the gamma counter. Likewise, a 100 μL bolus containing 730 kBq of either [^177^Lu]Lu-AAZTA^5^-LM4 or [^177^Lu]Lu-DOTA-LM3 and corresponding to 40 pmol AAZTA^5^-LM4 or DOTA-LM3 was iv injected in groups of four of inoculated mice. Animals were euthanized at 4, 24 and 48 h pi and the procedure described above was followed. Biodistribution data were calculated with the aid of suitable standards of the administered dose as percent of the injected activity per gram tissue (%IA/g) with excel software (Microsoft Corporation, Redmond, Washington, DC, USA). Results were provided as average ± sd values, *n* = 4 per time point, via the PRISM^TM^ 6.0 GraphPad software (San Diego, CA, USA). Statistical analysis was performed as previously described [30]. 

#### 2.3.3. [^111^In]In-DATA^5^-LM4 SPECT/CT in HEK293-SST_2_R/wtHEK293 Tumor-Bearing Mice

Twin HEK293-SST_2_R and wtHEK293 xenografts were developed in three mice as described above and a bolus of [^111^In]In-AAZTA^5^-LM4 (100 μL, 9–12 MBq corresponding to 3 nmol total AAZTA^5^-LM4 in vehicle: saline/EtOH 9/1 *v*/*v*) was injected via their tail vein. Mice were euthanized at 4 h (2 mice) and 24 h pi (1 mouse). Tomographic SPECT/CT imaging was conducted on the y-CUBE/x-CUBE systems (Molecubes, Ghent, Belgium) following a previously described protocol [45]. 

Animal experiments were performed according to European and national regulations in certified facilities (EL 25 BIO exp021). The study protocols were approved by the Department of Agriculture and Veterinary Service of the Prefecture of Athens (approval of stability studies: #1609, 24 April 2019—approval of biodistribution and imaging studies #1610, 24 April 2019).

### 2.4. Patient Study

A 68-year-old female patient with a gastric NET of WHO grade III and a Ki67 index >15% with multiple lymph node and hepatic metastases was included in this study following her signed written informed consent. Both PET/CT imaging of NETs with [^68^Ga]Ga-DATA^5m^-LM4 and the subsequent SPECT/CT of the radionuclide therapy study with [^177^Lu]Lu-AAZTA^5^-LM4 were conducted in compliance with the Declaration of Helsinki. They were approved by the Institutional Ethics Committee of All India Institute of Medical Sciences (Ref. No. IECPG-210, 24/03/22 IECPG-290, approval day 27 April 2022, and Ref. No. IECPG-343, approval day 28 April 2022, respectively). 

After a preceding PET/CT imaging with [^68^Ga]Ga-DOTA-NOC ([^68^Ga]Ga-DOTA-[1-Nal^3^]octreotide) [46], the patient underwent first PET/CT with [^68^Ga]Ga-DATA^5m^-LM4 on a dedicated GE Discovery 710 × 128 Slice PET/CT Scanner (GE HealthCare Technologies Inc.,/GE Healthcare, Chicago, IL, USA), with a 40 mm detector at a 0.35 s rotation speed and a 128-slice CT scanner. The images were corrected for random and scatter counts, decay correction and dead time correction. PET images were reconstructed with iterative reconstruction using ordered subset expectation maximization algorithm (OSEM) (2 iterations, 24 subsets). They were processed and analyzed with a dedicated commercially available workstation (GE Xeleris). Further details on the patient preparation, scan and data acquisition protocol are included in the Supplementary Materials.

Following injection of [^177^Lu]Lu-AAZTA^5^-LM4, whole-body scans were performed on a Dual Head Gamma Camera (GE Discovery NM/CT 670; GE HealthCare Technologies Inc.,/GE Healthcare, Chicago, IL, USA) using a high-energy general purpose (HEGP) collimator. Scans were acquired in the Lu-177 window at a 20% energy window with a peak at 208 keV and 113 keV. Serial whole-body scans were acquired at 4, 17–24, 48, 96, 120 and 168 h pi. The matrix size for the whole-body scan was 256 × 1024. Anterior and posterior views were acquired by using both detectors. After acquiring the whole-body scan, serial SPECT/CT of the region of interest, i.e., abdomen and pelvis area in our study were acquired at 4, 17–24, 48, 96, 120 and 168 h pi. The scans were acquired in H mode, with a starting angle of 0°. The matrix size used for the study was 128 × 128. The arc per detector was 180º and the number of views was 60. The scan mode was step and shoot, with a duration of 15 s/step. The CT scan involved a diagnostic dose CT with 300–380 mAs and 120 kVp, with a slice thickness of 2.5 mm and pitch of 0.6. The matrix size used for CT was 512 × 512. CT was used for attenuation correction as well as anatomical localization.

## 3. Results

### 3.1. Ligands and Radioligands

Analytical data for the new AAZTA^5^-LM4 bioconjugate are presented in Tables S1 and S2 (Supplementary Materials). Table S1 contains data of HPLC analyses in two separate systems and MALDI-TOF MS data. Table S2 includes results from amino acid analysis. All data turned out to be consistent with the formation of AAZTA^5^-LM4 in high purity (≥99%).

For the preclinical studies, both AAZTA^5^-LM4 and DOTA-LM3 were labeled with In-111 and Lu-177, as previously described [16,20,21,42]. Notably, radiolabeling of AAZTA^5^-LM4 with either radiometal proceeded by brief incubation at 50 °C (10 min for In-111 and 30 min for Lu-177). The final radiolabeled products were obtained in a ≥99% purity under these conditions at apparent molar activities of 3.7–7.4 MBq/nmol peptide for In-111 and 37 MBq/nmol peptide for Lu-177. Representative HPLC chromatograms for [^111^In]In-AAZTA^5^-LM4 and [^177^Lu]Lu-AAZTA^5^-LM4 are included in Figures S1a and S1b, respectively (Supplementary Materials). 

Interestingly, alternative radiolabeling protocols for labeling of AAZTA^5^-LM4 with Lu-177 and comprising manual (at room temperature) and semi-automated module-involving procedures (at 50 °C) were successfully conducted as well (Supplementary Materials). Results from a kinetic study of [^177^Lu]Lu-AAZTA^5^-LM4 formation at room temperature using different amounts of precursor (1–30 nmol) are summarized in Figure S2 (Supplementary Materials), showing rapid coordination of the radiometal. Results from [^177^Lu]Lu-AAZTA^5^-LM4 stability assessments for up to 10 d incubation at 37 °C in normal saline (NS), phosphate buffer saline (PBS) or human serum (HS) are reported as well (Figure S3). These data confirmed the high stability of the radioligand within the 10 d period in NS and PBS. In HS, however, a slow release of the radiometal was observed, reaching a maximum of 35% 10 d later. Such release was found to be negligible in the first 24 h of incubation. Finally, results from the quality control of module-synthesized [^177^Lu]Lu-AAZTA^5^-LM4, comprising radioanalytical HPLC and TLC methods are presented in Figure S4 (Supplementary Materials) and confirm the >99% purity radioligand production at an apparent molar activity of 40 MBq/nmol. Furthermore, results from the preparation and quality control of [^68^Ga]Ga-DATA^5m^-LM4 and [^177^Lu]Lu-AAZTA^5^-LM4 injected into the patient are reported in Supplementary Materials.

### 3.2. In Vitro Evaluation

#### 3.2.1. Affinity for the Human SST_2_R

**Figure 2:**
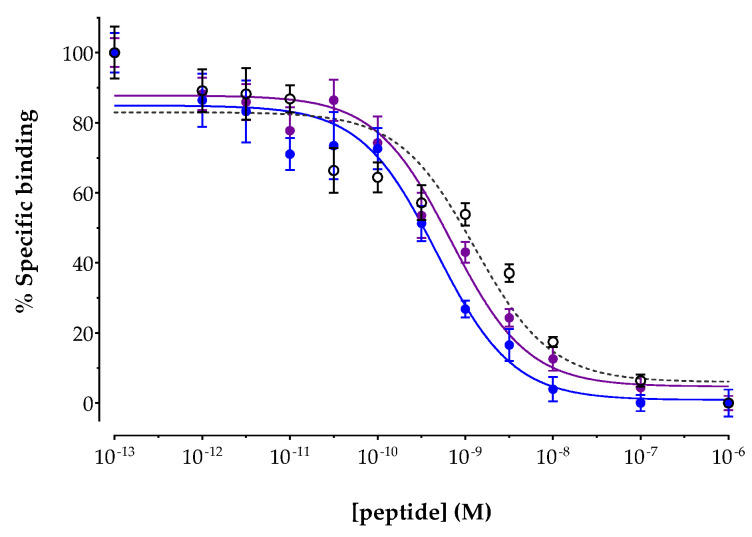
Displacement of [^125^Tyr^25^]LTT-SS28 from SST_2_R binding sites in HEK293-SST_2_R cell membranes by increasing concentrations of ○ AAZTA^5^-LM4 (IC_50_ = 1.69 ± 0.47 nM, *n* = 4); ● [^nat^In]In-AAZTA^5^-LM4 (IC_50_ = 0.45 ± 0.05 nM, *n* = 3) and ● [^nat^Lu]Lu-AAZTA^5^-LM4 (IC_50_ = 0.55 ± 0.38 nM, *n* = 3); results represent mean IC_50_ values ± sd, *n*: number of separate experiments in triplicate.

The affinities of AAZTA^5^-LM4 and its metal-tagged versions [^nat^In]In-AAZTA^5^-LM4 and [^nat^Lu]Lu-AAZTA^5^-LM4 were determined by competition binding assays against [^125^I-Tyr^25^]LTT-SS28 in freshly harvested HEK293-SST_2_R cell membranes. Results are included in Figure 2, showing the new analogs displacing [^125^I-Tyr^25^]LTT-SS28 from SST_2_R-binding sites in the membranes in a monophasic and dose-dependent manner. The IC_50_ values (mean ± sd) calculated for each individual analog were 1.69 ± 0.47 nM (*n* = 4) for AAZTA^5^-LM4, 0.45 ± 0.05 nM (*n* = 3) for [^nat^In]In-AAZTA^5^-LM4 and 0.55 ± 0.38 nM (*n* = 3) for [^nat^Lu]Lu-AAZTA^5^-LM4. It is interesting to note that coordination of either metal in AAZTA^5^-LM4 led to a significant increase in binding affinity (*p* < 0.01), with the affinities of [^nat^In]In-AAZTA^5^-LM4 and [^nat^Lu]Lu-AAZTA^5^-LM4 found comparable to each another (*p* > 0.05).

#### 3.2.2. Comparative Radioligand Uptake in HEK293-SST_2_R Cells

**Figure 3:**
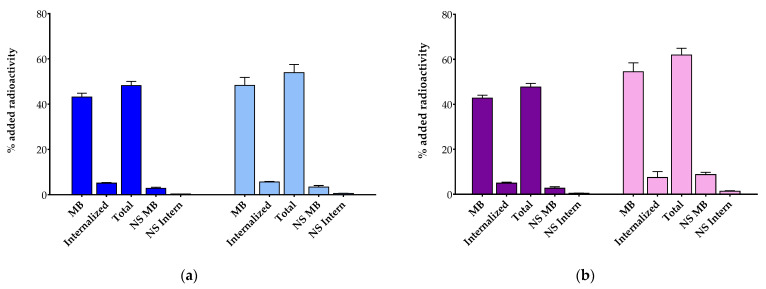
Radioligand uptake internalization during 1 h incubation at 37 °C in HEK293-SST_2_R cells. (**a**) ■, [^111^In]In-AAZTA^5^-LM4 and ■, [^111^In]In-DOTA-LM3; (**b**) ■, [^177^Lu]Lu-AAZTA^5^-LM4 and ■, [^177^Lu]Lu-DOTA-LM3; MB = specific membrane bound, Internalized = specific internalized; Total = specific MB + specific Internalized, NS MB = non-specific MB, NS Intern = non-specific internalized. Results were acquired from 3 independent experiments performed in triplicate.

The uptake and internalization of [^111^In]In-AAZTA^5^-LM4 and [^177^Lu]Lu-AAZTA^5^-LM4 were directly compared with the respective [^111^In]In-DOTA-LM3 and [^177^Lu]Lu-DOTA-LM3 references by 1 h radioligand incubation at 37 °C in HEK293-SST_2_R cells. Cumulative results are shown in Figure 3a for In-111 and 3b for Lu-177. The total specific cell uptake of [^111^In]In-AAZTA^5^-LM4 (48.2 ± 1.8% of added activity) was found lower than that of [^111^In]In-DOTA-LM3 (54.0 ± 3.6% of added activity; *p* < 0.0001). The same trend was observed for [^177^Lu]Lu-AAZTA^5^-LM4 (47.7 ± 1.6% of added activity) and [^177^Lu]Lu-DOTA-LM3 (61.9 ± 3.0% of added activity; *p* < 0.0001). Interestingly, the bulk of radioactivity for all tested analogs was found on the cell membrane, as for example in the case of [^111^In]In-AAZTA^5^-LM4 (43.1 ± 1.7% membrane-bound fragment vs. 5.1 ± 0.3% internalized fragment). This pattern of cell distribution was consistent with SST_2_R-antagonist behavior. In all cases, cell uptake was markedly reduced in the presence of excess TATE, in accordance with an SST_2_R-mediated process. 

### 3.3. Animal Studies

#### 3.3.1. In Vivo Metabolic Stability of [^111^In]In-AAZTA^5^-LM4 and [^177^Lu]Lu-AAZTA^5^-LM4

The two [^111^In]In-AAZTA^5^-LM4 and [^177^Lu]Lu-AAZTA^5^-LM4 radioligands were iv injected in healthy mice and blood samples were collected 5 min pi and aliquots of suitably processed blood samples were analyzed by radio HPLC. The absence of radiometabolites in the blood samples confirmed the high in vivo metabolic stability of the radioligands. The representative radiochromatogram for [^177^Lu]Lu-AAZTA^5^-LM4 is presented in Figure S5 (Supplementary Materials). 

#### 3.3.2. Biodistribution in Mice Bearing Twin HEK293-SST_2_R and wtHEK293 Xenografts

**Figure 4:**
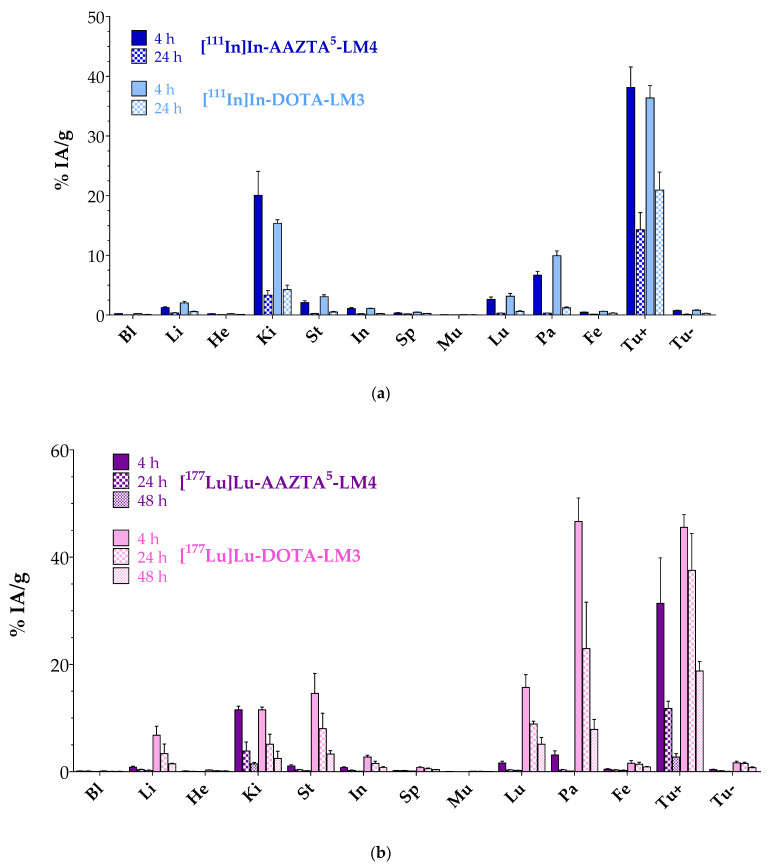
Comparative biodistribution data in SCID mice bearing twin HEK293-SST_2_R and wtHEK293 xenografts. (**a**) ■, [^111^In]In-AAZTA^5^-LM4 and ■, [^111^In]In-DOTA-LM3; (**b**) ■, [^177^Lu]Lu-AAZTA^5^-LM4 and ■, [^177^Lu]Lu-DOTA-LM3; solid bars for 4 h pi, checkered bars for 24 h pi and crossed bars for 48 h pi. Data are expressed as %IA/g and shown as average values ± sd, *n* = 4; Bl: blood, Li: liver, He: heart, Ki: kidneys, St: stomach, In: intestines, Sp: spleen, Mu: muscle, Lu: lungs, Pa: pancreas, Fe: femur, Tu+: HEK293-SST_2_R tumor, Tu-: wtHEK293 tumor.

Comparative biodistribution results of [^111^In]In-AAZTA^5^-LM4 and [^111^In]In-DOTA-LM3 at 4 and 24 h pi in SCID mice bearing double HEK293-SST_2_R and wtHEK293 xenografts are included in Figure 4a and in numerical values in Table S3 (Supplementary Materials). Data are given as %IA/g and represent mean values ± sd, *n* = 4 per group. A high uptake was attained by both radioligands in the HEK293-SST_2_R but not in the SST_2_R-negative wtHEK293 tumors, implying a receptor-mediated process. For example, the HEK293-SST_2_R tumor uptake of [^111^In]In-AAZTA^5^-LM4 (38.13 ± 3.44%IA/g at 4 h pi and 14.28 ± 2.88%IA/g at 24 h pi) was found to be significantly lower in the wtHEK293 tumors (0.74 ± 0.05%IA/g at 4 h pi, *p* < 0.0001; and 0.13 ± 0.03%IA/g at 24 h pi, *p* < 0.0001). It is interesting to note that both compounds displayed comparable uptake in the SST_2_R-positive xenografts at 4 h pi ([^111^In]In-AAZTA^5^-LM4: 38.13 ± 3.44%IA/g; [^111^In]In-DOTA-LM3: 36.38 ± 2.05%IA/g; *p* > 0.05), which, however, declined over time for both, albeit not equally fast. Thus, at 24 h pi, [^111^In]In-DOTA-LM3 turned out to retain a higher tumor uptake (20.93 ± 3.02%IA/g) than [^111^In]In-AAZTA^5^-LM4 (14.28 ± 2.88%IA/g; *p* < 0.0001). On the other hand, uptake and retention in the background differed between the two radioligands. Accordingly, [^111^In]In-AAZTA^5^-LM4 displayed lower values in the SST_2_R-expressing organs, especially in the pancreas (0.31 ± 0.05%IA/g at 24 h pi; Tu-to-Pa: 46.1) vs. [^111^In]In-DOTA-LM3 (1.20 ± 0.14%IA/g, *p* < 0.0001; Tu-to-Pa: 17.44), which further declined over time, consistent with radiolabeled antagonists. Kidney values were initially higher for [^111^In]In-AAZTA^5^-LM4, but reached similar levels at 24 h pi, yielding comparable tumor-to-kidney ratios ([^111^In]In-AAZTA^5^-LM4: 1.9 at 4 h and 4.3 at 24 h; [^111^In]In-DOTA-LM3: 2.4 at 4 h and 4.9 at 24 h). 

Comparative biodistribution results for [^177^Lu]Lu-AAZTA^5^-LM4 and [^177^Lu]Lu-DOTA-LM3 at 4, 24 and 48 h pi in the same mice model are included in Figure 4b and in numerical values in Table S4 (Supplementary Materials). It is quite apparent that differences in the biodistribution patterns between [^177^Lu]Lu-AAZTA^5^-LM4 and [^177^Lu]Lu-DOTA-LM3 were much more pronounced than between the respective In-111 radioligands. Along these lines, the uptake of [^177^Lu]Lu-AAZTA^5^-LM4 in the HEK293-SST_2_R xenografts was significantly lower (e.g., 31.38 ± 4.24%IA/g at 4 h pi; 2.73 ± 0.33%IA/g at 48 h pi) in comparison with [^177^Lu]Lu-DOTA-LM3 at all tested time intervals (e.g., 45.55 ± 1.66%IA/g at 4 h pi, *p* < 0.0001; 18.76 ± 0.88%IA/g at 48 h pi, *p* < 0.0001). The uptake of [^177^Lu]Lu-AAZTA^5^-LM4 and [^177^Lu]Lu-DOTA-LM3 in the SST_2_R-negative tumors was minimal at all time points, once again confirming an SST_2_R-mediated mechanism. Of particular interest is the fact that the higher tumor uptake of [^177^Lu]Lu-DOTA-LM3 was found compromised by the unfavorably high radioactivity levels in most physiological organs, such as the pancreas, stomach, lungs, liver and intestines, when compared with [^177^Lu]Lu-AAZTA^5^-LM4 at all time points. For example, pancreatic uptake was markedly higher for [^177^Lu]Lu-DOTA-LM3 (46.62 ± 2.20%IA/g at 4 h pi, Tu-to-Pa: 2.0; 7.85 ± 0.94%IA/g at 48 h pi, Tu-to-Pa: 2.4) than for [^177^Lu]Lu-AAZTA^5^-LM4 (3.10 ± 0.39%IA/g at 4 h pi, *p* < 0.0001, Tu-to-Pa: 10.1; 0.13 ± 0.01%IA/g at 48 h pi, *p* < 0.0001, Tu-to-Pa: 21) with the Tu-to-Pa ratios much more in favor of the new radioligand. Kidney uptake, however, represented an exception, with uptake values found comparable for the two agents, thereby resulting in more favorable Tu-to-Ki ratios for the [^177^Lu]Lu-DOTA-LM3 reference (11.52 ± 0.26%IA/g at 4 h pi, Tu-to-Ki: 2.7; 2.47 ± 0.67%IA/g at 48 h pi, Tu-to-Ki: 2.0) compared with [^177^Lu]Lu-AAZTA^5^-LM4 (11.55 ± 0.34%IA/g at 4 h pi, *p* > 0.05, Tu-to-Ki: 3.9; 1.45 ± 0.13%IA/g at 48 h pi, *p* > 0.05, Tu-to-Ki: 7.6). 

#### 3.3.3. SPECT/CT of Mice Bearing Twin HEK293-SST_2_R and wtHEK293 Xenografts

**Figure 5:**
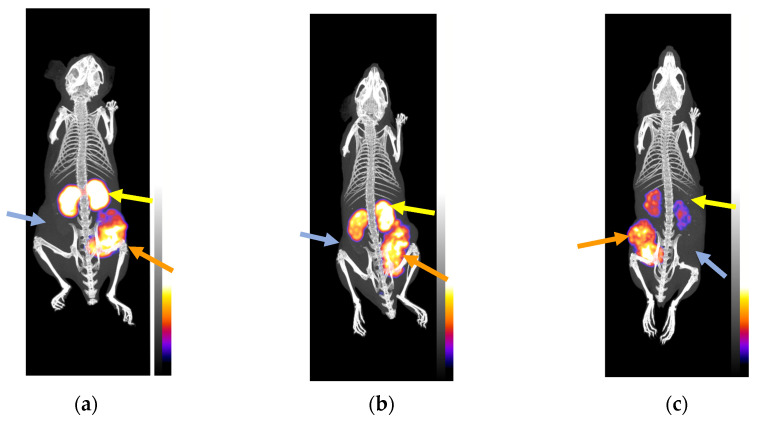
Static whole-body SPECT/CT images of SCID mice bearing twin HEK293-SST_2_R and wtHEK293 tumors in their flanks at (**a**) and (**b**) 4 h pi and (**c**) at 24 h pi of [^111^In]In-AAZTA^5^-LM4; orange arrows are pointing to HEK293-SST_2_R xenografts and light blue arrows to wtHEK293 tumors, devoid of SST_2_R expression. Intense uptake is observed in the SST_2_R-expressing tumors but no uptake is evident in the negative controls. Yellow arrows are directed toward the kidneys; the initial kidney uptake at (**a**) and (**b**) 4 h pi notably declines at (**c**) 24 h pi. The color bars indicate the difference in accumulated activity (purple being the lowest and white the highest level of accumulation).

SPECT/CT was performed for [^111^In]In-AZTA^5^-LM4 in three SCID mice bearing double HEK293-SST_2_R and wtKEK293 tumors in their flanks at 4 (two animals) and 24 h pi (one mouse); images are depicted in Figure 5. [^111^In]In-AZTA^5^-LM4 was selectively taken up only by the SST_2_R-expressing tumors, but not by the receptor-negative controls, verifying an SST_2_R-mediated process in agreement with biodistribution findings. Kidney uptake was intensive at 4 h pi, but significantly declined at 24 h pi, leading to favorable increase in Tu-to-Ki ratios concordant with biodistribution results.

### 3.4. Patient Study

**Figure 6:**
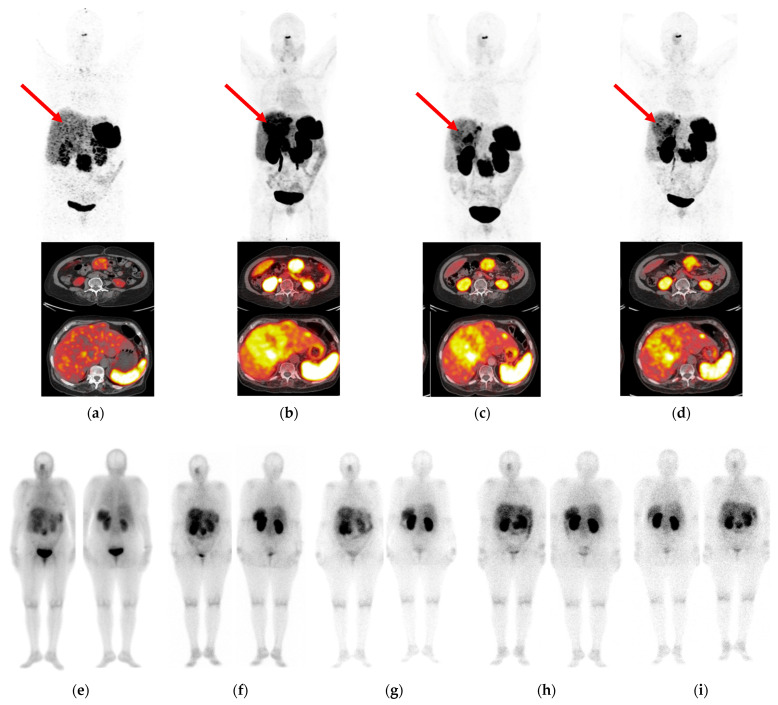
Correlation of PET/CT imaging (**a**–**d**) with SPECT/CT imaging (**e**–**i**) in a 68-year-old female patient with a stomach NET with multiple lymph node and liver metastases. (**a**) Mild to negligible radiotracer concentration in pathological lesions on PET/CT with [^68^Ga]Ga-DOTA-NOC at 1 h pi (reference) vs. PET/CT with [^68^Ga]Ga-DATA^5m^-LM4 at (**b**) 10 min pi, (**c**) 1 h pi and (**d**) 3 h pi, including whole-body and transaxial views. As indicated by the red arrows, mild to negligible uptake was displayed by [^68^Ga]Ga-DOTA-NOC in the liver metastases, whereas the uptake of [^68^Ga]Ga-DATA^5m^-LM4 was notably superior. The same patient was selected for sequential SPECT/CT imaging with [^177^Lu]Lu-AAZTA^5^-LM4 at (**e**) 4 h, (**f**) 17 h, (**g**) 24 h, (**h**) 48 h and (**i**) 72 h pi (anterior/posterior views included for each time point).

First impressions from the tumor targeting and pharmacokinetic behavior of [^177^Lu]Lu-AAZTA^5^-LM4 were acquired in a 68-year-old female patient with advanced stomach NET with extensive spread to the liver and lymph nodes. Representative imaging results are shown in Figure 6.

The patient initially underwent PET/CT with [^68^Ga]Ga-DOTA-NOC [46], which revealed weak uptake in the liver metastases. PET/CT imaging with the SST_2_R-antagonist [^68^Ga]Ga-DATA^5m^-LM4 was much more successful in the visualization of hepatic metastases as early as 10 min pi and spanning up to 3 h pi. This clearly advantageous performance of the [^68^Ga]Ga-DATA^5m^-LM4 antagonist compared to the [^68^Ga]Ga-DOTA-NOC agonist was the impetus to explore the overall biodistribution of the respective [^177^Lu]Lu-AAZTA^5^-LM4 in this patient applying sequential SPECT/CT imaging in a period of 4 h–72 h pi. At this early stage of the study, the priority was set to explore the uptake and retention of the new antagonist in the lesions, a critical issue for its value and applicability for therapy. In parallel with the exceptional results of [^68^Ga]Ga-DATA^5m^-LM4 PET/CT, the new radioligand [^177^Lu]Lu-AAZTA^5^-LM4 displayed high tumor uptake, which peaked at 17 h pi. Some uptake was observed in respiratory mucosa, spleen and kidneys, with rather low uptake in the liver. Radioactivity was excreted predominantly via the urinary system, with some portion of hepatobiliary excretion being observed. Accordingly, the highest tumor-to-background ratio was achieved at 24 h pi. These preliminary clinical results need to be confirmed by more patient cases, and careful dosimetric studies must be conducted prior to establishing the clinical value of [^177^Lu]Lu-AAZTA^5^-LM4 as a useful radionuclide therapy candidate in NET patients.

## 4. Discussion

We recently reported on [^68^Ga]Ga-DATA^5m^-LM4, a new SST_2_R-antagonist radiotracer for application in the diagnosis of human NETs with PET/CT [30]. Coupling of the hybrid chelator DATA^5m^ at the N-terminus of LM4 allowed for fast incorporation of radiogallium (Ga-67 or Ga-68) at conveniently lower temperatures than those required for DOTA-modified vectors, such as DOTA-LM3. Thus, [^67^Ga]Ga-DATA^5m^-LM4 was easily accessible and showed exceptional preclinical features in HEK293-SST_2_R/wtHEK293 cells and tumor-bearing mice when compared with [^67^Ga]Ga-DOTA-LM3. Moreover, in a first proof-of-principle study in a NET patient, [^68^Ga]Ga-DATA^5m^-LM4 was able to visualize tumor lesions accurately and with a high contrast on PET/CT. We have now attached the AAZTA^5^-chelator in place of DATA^5m^ to LM4 to allow for facile labeling with a broader palette of trivalent metals of medical interest [34]. According to recent reports, AAZTA^5^-derivatized vectors could be successfully labeled with Lu-177, Sc-44 or In-111 at lower temperatures. The subsequent study of resulting analogs has brought to light the easiness of AAZTA^5^ methodology to provide ready-to-use radioligands in a clinical setting [33,35,38,39,40,41].

In the present work, AAZTA^5^-LM4 and DOTA-LM3 were labeled with In-111 and Lu-177 and the biological profiles or forming radiopeptides were directly compared in HEK293-SST_2_R/wtHEK293 cells and tumors thereof raised in mice. Typically, higher and longer heating was required for full incorporation of In-111 and Lu-177 by DOTA-LM3 (80 °C for 30 min), as opposed to AAZTA^5^-LM4, which could be labeled at less drastic conditions (50 °C for 10 min for In-111, 30 min for Lu-177). It should be noted that equally successful labeling of AAZTA^5^-LM4 with Lu-177 could be achieved manually at room temperature or via a module semi-automatic process preset at 50 °C. Exceptional reproducibility was confirmed across these methods, whereby [^177^Lu]Lu-AAZTA^5^-LM4 could be easily obtained in >98% radiochemical purity at a molar activity of 40 MBq/nmol. 

It is interesting to observe that the SST_2_R binding affinity of AAZTA^5^-LM4 (IC_50_ = 1.69 ± 0.47 nM) slightly improved after coordination of indium ([^nat^In]In-AAZTA^5^-LM4 IC_50_ = 0.45 ± 0.05 nM) or lutetium ([^nat^Lu]Lu-AAZTA^5^-LM4 IC_50_ = 0.55 ± 0.38 nM). These values fall well within the range previously determined by the same assay for DATA^5m^-LM4 (IC_50_ = 1.24 ± 0.20 nM) and [^nat^Ga]Ga-DATA^5m^-LM4 (IC_50_ = 1.61 ± 0.32 nM) [30]. It should be noted that previous studies with DOTA-LM3 (IC_50_ = 1.4 ± 0.5 nM) revealed striking changes in SST_2_R affinities upon coordination of gallium (IC_50_ = 12.5 ± 0.4.3 nM), but not lutetium (IC_50_ = 1.6 ± 0.3 nM); further changes were observed as well by switching the chelator from DOTA to NODAGA in LM3 [16,47,48]. Thus, a strong impact of metal, chelator or metal–chelate on SST_2_R affinity was evident in the case of LM3, with such differences being practically absent in DATA^5m^/AAZTA^5^-LM4 and their metal-tagged versions. [^111^In]In-AAZTA^5^-LM4 and [^111^In]In-DOTA-LM3 displayed comparable uptake in HEK293-SST_2_R cells, whereas [^177^Lu]Lu-DOTA-LM3 displayed clearly more pronounced cell uptake than [^177^Lu]Lu-AAZTA^5^-LM4 (Figure 3). Interestingly, [^67^Ga]Ga-DATA^5m^-LM4 had previously demonstrated much superior uptake compared with [^67^Ga]Ga-DOTA-LM3, which was poorly taken up in the same cells [30]. This discrepancy is consonant with SST_2_R affinity differences and further underscores the impact of metal–chelate on the biological responses of LM3 radioligands. It should be stressed that in all cases, the bulk of radioactivity was found attached to the cell membrane, with only a small fraction being internalized, consonant with an SST_2_R-antagonist profile.

After injection in animals, both [^111^In]In-AAZTA^5^-LM4 and [^177^Lu]Lu-AAZTA^5^-LM4 were found to be metabolically robust, similarly to [^67^Ga]Ga-DATA^5m^-LM4 [30]. It appears that LM4-based radioligands withstand the degrading action of major proteolytic enzymes, such as neutral endopeptidase or angiotensin-converting enzyme, implicated in the rapid catabolism of many radiopeptides in vivo and compromising their delivery to tumor sites [49]. The biodistribution profiles of [^111^In]In-AAZTA^5^-LM4 and [^177^Lu]Lu-AAZTA^5^-LM4 were subsequently compared side by side with the respective [^111^In]In-DOTA-LM3 and [^177^Lu]Lu-DOTA-LM3 references. Interestingly, few differences were observed in the biodistributions of [^111^In]In-AAZTA^5^-LM4 and [^111^In]In-DOTA-LM3 in mice bearing twin HEK293-SST_2_R/wtHEK293 xenografts (Figure 4a). In line with their comparable in vitro uptake in HEK293-SST_2_R cells, the uptake of the two radiotracers in the HEK293-SST_2_R xenografts was also similar. Tumor retention between 4 and 24 h pi was found to be better for [^111^In]In-DOTA-LM3, but on the other hand, background levels dropped faster for [^111^In]In-AAZTA^5^-LM4. Eventually, tumor-to-background ratios turned out to be almost identical for [^111^In]In-AAZTA^5^-LM4 and [^111^In]In-DOTA-LM3 and were found to be improving with time. This positive result could be visualized for [^111^In]In-AAZTA^5^-LM4 on small-animal SPECT/CT (Figure 5). In comparison with [^67^Ga]Ga-DATA^5m^-LM4 and [^67^Ga]Ga-DOTA-LM3, previously assessed as PET/CT radiotracers in the same animal model [30], we observe that the respective two In-111 radiotracers tested herein displayed higher tumor uptake and much lower renal retention. Interestingly, the most striking differences were found between [^111^In]In-DOTA-LM3 and [^67^Ga]Ga-DOTA-LM3 for the tumor (In: 36.38 ± 2.05%IA/g and Ga: 16.83 ± 1.22%IA/g) and kidneys (In: 15.36 ± 0.58%IA/g and Ga: 37.65 ± 3.44%IA/g) at 4 h pi. These results favor a future application of [^111^In]In-AAZTA^5^-LM4, and possibly also [^111^In]In-DOTA-LM3, as alternative diagnostic tracers for SPECT/CT, by allowing for imaging at later time points associated with higher tumor-to-background ratios. Furthermore, they offer the option of SST_2_R-targeted diagnosis of NETs in nuclear medicine facilities lacking PET/CT instrumentation. 

Likewise, the biodistribution of [^177^Lu]Lu-AAZTA^5^-LM4 and [^177^Lu]Lu-DOTA-LM3 were directly compared in the same twin HEK293-SST_2_R/wtHEK293 tumor-bearing animal model (Figure 4b). In line with in vitro cell uptake findings, [^177^Lu]Lu-DOTA-LM3 displayed higher uptake in the HEK293-SST_2_R xenografts compared with [^177^Lu]Lu-AAZTA^5^-LM4 at all time points tested. However, the background radioactivity levels were unfavorably much higher for [^177^Lu]Lu-DOTA-LM3 in almost all organs and tissues, except for the kidneys. It is not easy to predict to what extent this pattern could be attributed to affinity differences between the mice and human receptors, or if it can be altered by the administration of higher peptide amounts or kidney protection regimens, or if it can be extrapolated from mice to humans. Further dedicated studies are warranted to properly address these questions, which are essential for dosimetry calculations and rational therapy planning. 

In a first step toward this goal, we then investigated the biodistribution of [^177^Lu]Lu-AAZTA^5^-LM4 in a patient with a previously confirmed gastric NET metastasized in the liver and lymph nodes. Initial PET/CT with the established SST_2_R-agonist [^68^Ga]Ga-DOTA-NOC [46] failed to accurately reveal most liver lesions as opposed to PET/CT with the SST_2_R-antagonist [^68^Ga]Ga-DATA^5m^-LM4. The latter successfully visualized hepatic lesions with high contrast, confirming previous reports on the superior diagnostic power of SST_2_R-antagonists compared with agonists [10,12,13,14,15]. Based on this promising outcome, the patient was next injected with [^177^Lu]Lu-AAZTA^5^-LM4 and followed by SPECT/CT for tumor uptake and retention and overall tissue distribution pattern up to 72 h pi. The radioligand was well tolerated by the patient, showing high and prolonged uptake in tumor lesions. In contrast, the radioactivity declined faster from physiological tissues, leading to favorable tumor-to-background ratios at 24 h pi. Excretion was mainly achieved via the urinary system, with the kidneys still visible at 72 h pi. The overall pattern of [^177^Lu]Lu-AAZTA^5^-LM4 in the patient turned out to be quite promising for radionuclide therapy, after further study of human dosimetry, safety, therapeutic efficacy and selection of a suitable therapeutic scheme. Ongoing studies will eventually establish its actual therapeutic value in the clinic.

## 5. Conclusions

Derivatization of the SST_2_R-antagonist LM4 with the hybrid chelator AAZTA^5^ has allowed convenient labeling with In-111 (for diagnostic SPECT/CT) and Lu-177 (for radionuclide therapy), expanding the application prospects of LM4 beyond [^68^Ga]Ga-DATA^5m^-LM4 PET/CT, previously proposed. The hybrid character of DATA^5m^/AAZTA^5^ chelators allows for faster coordination of the pertinent radiometals under milder conditions than usual, most convenient in a clinical environment. Thus, the preclinical evaluations of [^111^In]In-AAZTA^5^-LM4 and [^177^Lu]Lu-AAZTA^5^-LM4 were investigated herein in HEK293-SST_2_R cells and double HEK293-SST_2_R/wtHEK293 tumor-bearing mice in direct comparison with the respective [^111^In]In-DOTA-LM3 and [^177^Lu]Lu-DOTA-LM3 references. This study has revealed the exceptional qualities of the LM4 analogs for further assessment in humans. In a first proof-of-principle study performed in a metastatic NET patient, [^177^Lu]Lu-AAZTA^5^-LM4 showed sustained tumor uptake up to 72 h pi versus a faster decline of background radioactivity and was well tolerated by the patient. Further studies are warranted to explore the applicability of [^177^Lu]Lu-AAZTA^5^-LM4 in the treatment of SST_2_R-positive human NETs based on previous [^68^Ga]Ga-DATA^5m^-LM4 PET/CT.

## Data Availability

Not applicable.

## Supplementary Materials

The following supporting information can be downloaded at: https://www.mdpi.com/article/10.3390/pharmaceutics15030776/s1, Figure S1: Radiochromatograms of [^111^In]In-AAZTA^5^-LM4 and [^177^Lu]Lu-AAZTA^5^-LM4-labeled products; Figure S2: Kinetics of [^177^Lu]Lu-AAZTA^5^-LM4 formation; Figure S3: Stability of [^177^Lu]Lu-AAZTA^5^-LM4 during incubation in NS, PBS and HS at 37 °C; Figure S4: Quality control results of [^177^Lu]Lu-AAZTA^5^-LM4 from labeling via the module with HPLC radiochromatogram and TLC footprints; Figure S5: Metabolic stability of [^111^In]In-AAZTA^5^-LM4 and [^177^Lu]Lu-AAZTA^5^-LM4 during HPLC analysis of peripheral mice blood samples; Tables S1/S2: Analytical data/amino acid analysis of AAZTA^5^-LM4; Table S3: Biodistribution of [^111^In]In-AAZTA^5^-LM4 and [^111^In]In-DOTA-LM3 in twin HEK293-SST_2_R/wtHEK293 tumor-bearing mice (numerical values); Table S4: Biodistribution of [^177^Lu]Lu-AAZTA^5^-LM4 and [^177^Lu]Lu-DOTA-LM3in twin HEK293-SST_2_R/wtHEK293 tumor-bearing mice (numerical values).
