# Supplementary material for: [111In]In/[177Lu]Lu-AAZTA5-LM4 SST2R-Antagonists in Cancer Theranostics: From Preclinical Testing to First Patient Results
Source: Pharmaceutics. 2023 Feb 26;15(3):776. doi: 10.3390/pharmaceutics15030776 (PMC10053881; doi:10.3390/pharmaceutics15030776)
Supplement: Supplementary file 1 [file pharmaceutics-15-00776-s001.zip › pharmaceutics-2248405-supplementary.pdf]

pharmaceutics

MDPI

Article

Supplementary Materials: [111In]In/[177Lu]Lu-AAZTA5-LM4 SST2R-Antagonists in Cancer Theranostics: From Preclinical Testing to First Patient Results

Berthold A. Nock [1], Panagiotis Kanellopoulos [1], Euy Sung Moon [2], Maritina Rouchota [3], George Loudos [3], Sanjana Ballal [4], Madhav P. Yadav [4], Chandrasekhar Bal [4], Prashant Mishra [4], Parvind Sheokand [4], Frank Roesch [2], and Theodosia Maina [1,*]

Analytical data for AAZTA5-LM4

Analytical data for AAZTA5-LM4 (AAZTA5-p-Cl-Phe-c[DCys-4Pal-DAph(Cbm)-Lys-Thr-Cys]-DTyr-NH_2; Peptide Specialty Laboratories GmbH, Heidelberg, Germany), comprising purity via HPLC analysis and MALDI-TOF results are included in Table S1. Findings from amino acid analysis of AAZTA5-LM4 are presented in Table S2.

Table S1. Analytical data for AAZTA5-LM4.[a]

	HPLC		MW [d] calcd	MW found [e], m/z
	t_R (min)	% Purity		
system 2 [b]	20.6	>98%	1579.6	1578.6
system 3[c]	3.4	>98%		

[a] AAZTA5: 1,4-bis(carboxymethyl)-6-[bis (carboxymethyl)]amino-6-[pentanoic-acid]perhydro-1,4-diazepine), 4Pal; (4-pyridyl)alanine, DAph(Cbm): D-4-(carbamoyl)amino-phenylalanine; [b] system 2: HPLC with PDA-UV detection mode (Waters, Vienna, Austria) using a Waters XSelect CSH™ C18 reverse phase column (5 μm, 4.6 mm × 150 mm) eluted at a 1.0 mL/min flow rate with the following linear gradient: from 10% B/90% A to 15% B/85% A in 5 min, followed by a further 0.5%/min increase of B in the next 60 min (A = 0.01% TFA in H_2O (v/v) and B = MeCN); [c] system 3: HPLC on a Gilson Device (Middleton, WI/USA) using a Kinetex EVO C18 column (5 μm, 100 Å; Phenomenex, Torrance, CA/USA) eluted at a flow rate of 2 mL/min with the following gradient: 1 - 7.5 min 0% B/100% A – 75% B/25% A, whereby: 0.1% aq. TFA and B: 80% MeCN in A; [d] exact mass; [e] verification on MALDI TOF mass spectrometry (Ultraflex III, Bruker; Bremen, Germany).

Table S2. Results from the Amino Acid Analysis of AAZTA5-LM4.

Code	#	Name	Code	#	Name
A	0	Alanine	N	0	Asparagine
B	1	4Pal [a]	O	0	
C	2	Cysteine	P	0	Proline
D	0	Aspartic Acid	Q	0	Glutamine
E	0	Glutamic Acid	R	0	Arginine
F	0	Phenylalanine	S	0	Serine
G	0	Glycine	T	1	Threonine
H	0	Histidine	U	1	D-Aph(Cpm) [b]
I	0	Isoleucine	V	0	Valine
J	0		W	0	Tryptophane
K	1	Lysine	X	1	Cpa [c]
L	0	Leucine	Y	1	Tyrosine
M	0	Methionine	Z	0	

[a] 4Pal; (4-pyridyl)alanine; [b] DAph(Cbm): D-4-(carbamoyl)amino-phenylalanine; [c] Cpa: p-Cl-phenylalanine

Quality Control of [^{111}In]In-AAZTA^5-LM4 and [^{177}Lu]Lu-AAZTA^5-LM4 Used in Preclinical Studies

A typical radiochromatogram of HPLC analysis for the quality control of [^{111}In]In-AAZTA^5-LM4 and [^{177}Lu]Lu-AAZTA^5-LM4 labeled products prepared for preclinical testing is presented in Figure S1, (a) and (b), respectively. Labeling protocols and HPLC details (system 1) have been reported in the manuscript.

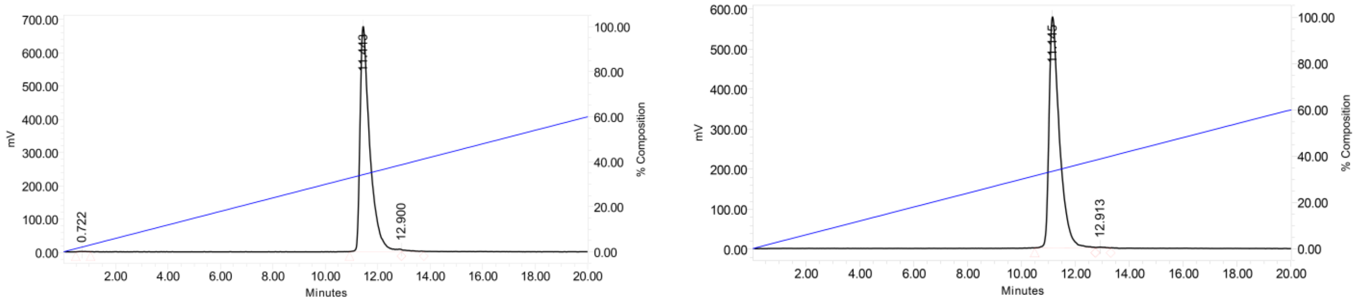

(**a**) (**b**)

Figure S1. Representative radiochromatogram of (**a**) [^{111}In]In-AAZTA^5-LM4 and (**b**) [^{177}Lu]Lu-AAZTA^5-LM4 labeled products; a >99% radiochemical purity could be confirmed for both radioligands; retention time (t_R) for [^{111}In]In-AAZTA^5-LM4 = 11.45 min and for [^{177}Lu]Lu-AAZTA^5-LM4 = 11.14 min; applying system 1.

Radiolabeling Study on [^{177}Lu]Lu-AAZTA^5-LM4

Additional results for labeling AAZTA^5-LM4 with Lu-177 were conducted, whereby the precursor (1 – 30 nmol, 1.58 – 47.4 µg) was added to a 1 M ammonium acetate solution (pH 5.5; 300 µL) followed by Lu-177 (90-100 MBq) and the mixture was shaken at ambient temperature (25 °C). Aliquots thereof were taken at 1, 3, 5, 10, 15, 30, 60 min and tested for [^{177}Lu]Lu-AAZTA^5-LM4 formation. Results of this study are presented below in Figure S2 and confirm fast coordination of the radiometal (already in 1 min) using as low as 1 nmol precursor at room temperature.

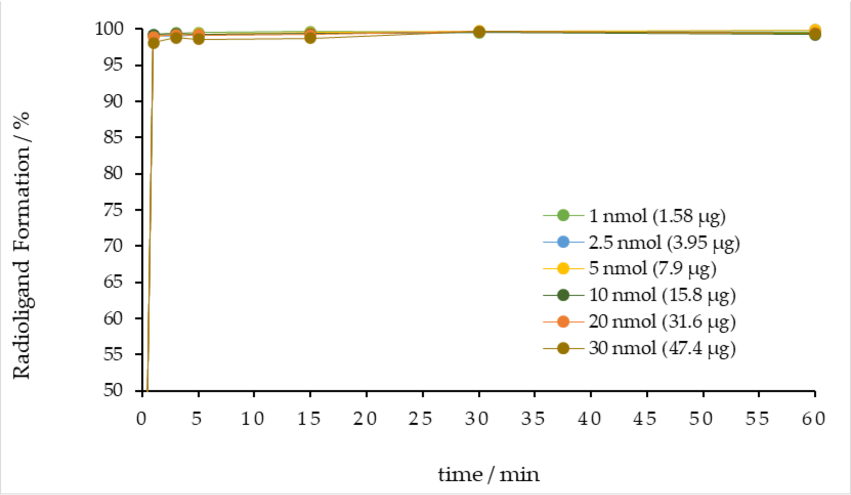

Figure S2. Kinetics of [^{177}Lu]Lu-AAZTA^5-LM4 formation using increasing amounts of precursor at 25 °C.

Study on [^{177}Lu]Lu-AAZTA^5-LM4 Stability

A sample (5 MBq) of the above labeling reaction mixture was taken after 1 h incubation and was mixed with either normal saline (NS), phosphate buffer saline (PBS) or human serum (HS) and was incubated at 37 °C in triplicate (n = 3). Aliquots thereof were collected at 1, 2, 4, 24, 48, 72, 144, 168, 240 h and analyzed for [^{177}Lu]Lu-AAZTA^5-LM4 stability. Results are summarized in Figure S3, below.

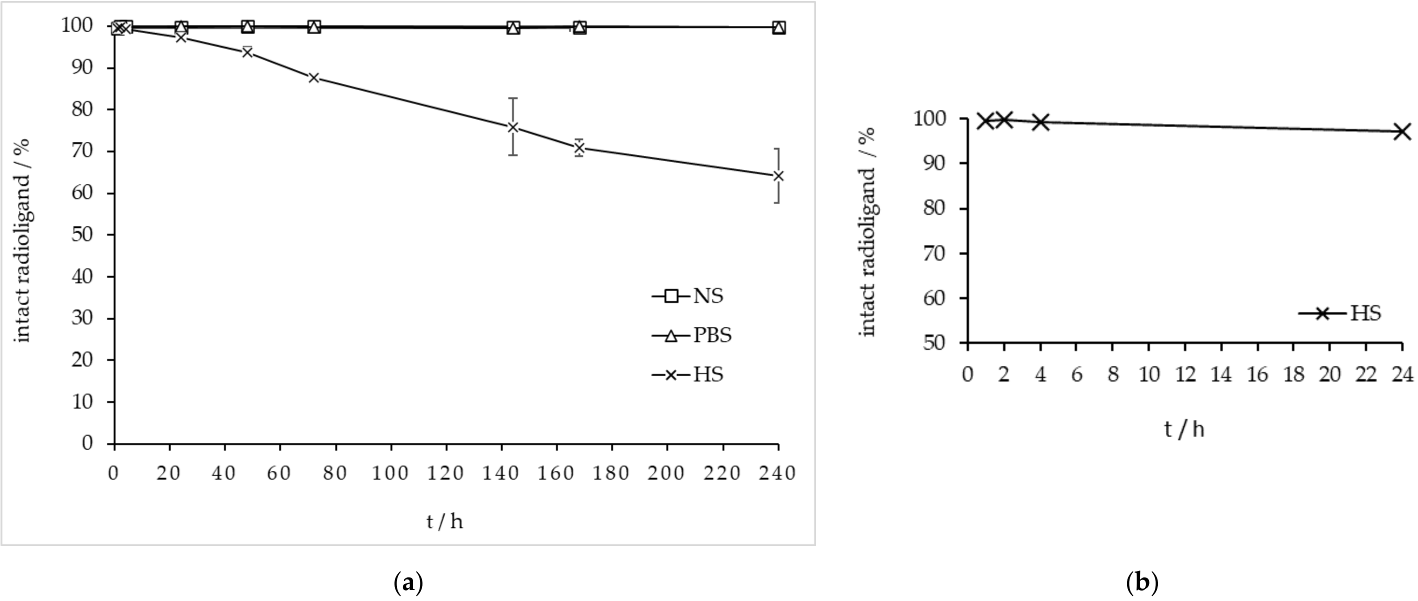

Figure S3. Stability of [^{177}Lu]Lu-AAZTA^5-LM4 during incubation in □ NS, △ PBS and × HS at 37 °C in (**a**) a period of 1-240 h and (**b**) in × HS in a period of 1-24 h.

Results reveal a high stability of the [^{177}Lu]Lu-AAZTA^5-LM4 in NS and PBS with a >98% Lu-177 coordinated in the AAZTA^5-chelator of the radioligand for as long as 10 days incubation at 37 °C. In HS however Lu-177 was found to be released from the metal chelate over time, with such release reaching up to 35% at day 10 (Figure S3 – (a)). Interestingly though very little radiometal release was evident within a 24 h time-frame (Figure S3 – (b)).

Radiosynthesis of [^{177}Lu]Lu-AAZTA^5-LM4 on the Module

Alternatively, radiolabeling of AAZTA^5-LM4 with Lu-177 was conducted semi-automatically using a module (Gaia Luna, Elysia-Raytest; Straubenhardt, Germany). Typically, Lu-177 (1472 MBq) were incubated for 40 min at 50 °C with the precursor (36.8 nmol, 58.1 µg) in a total volume of about 11 mL for a >99% radiometal incorporation at an apparent molar activity of 40 MBq/nmol.

Quality Control of Module-Produced [^{177}Lu]Lu-AAZTA^5-LM4

Quality control of the [^{177}Lu]Lu-AAZTA^5-LM4 produced via the Module method comprised radio-HPLC and radio-TLC analysis. HPLC analysis was conducted on a 7000 series Hitachi LaChrom system (Hitachi High-Tech Analytical Science Ltd.; Abingdon, UK), whereby a Phenomenex Luna C18 column (5 µm, 250 mm × 4.6 mm) was eluted at a flow rate of 1 mL/min with the gradient: from 90% A/10% B to 5% A/95% B in 10 min, whereby A = 0.1% TFA in water and B = 0.1% TFA in MeCN (t_R = 9.4 min). Twin TLC plates (Kieselgel 60 F254 coated aluminum plates, Merck Chemicals GmbH, Darmstadt, Germany) were developed in parallel with either Profile 1: 0.1 M citrate pH 4 – R_f [^{177}Lu]Lu-AAZTA^5-LM4 = 0, or Profile 2: 0.25 M ammonium acetate pH 4 /MeOH

(1/1) – R_f [^{177}Lu]Lu-AAZTA^5-LM4 = 9-10. The strips were scanned using a TLC imager (CR-35 Bio Test-Imager, DÜRR NDT GmbH & Co. KG; Bietigheim-Bissingen, Germany) with the aid of analysis software AIDA Elysia-Raytest (Straubenhardt, Germany). Representative results from HPLC analysis and TLC (Profile 1 and 2) are presented in Figure S4 (a), (b) and (c), respectively, confirming the excellent quality of the radiolabeled product.

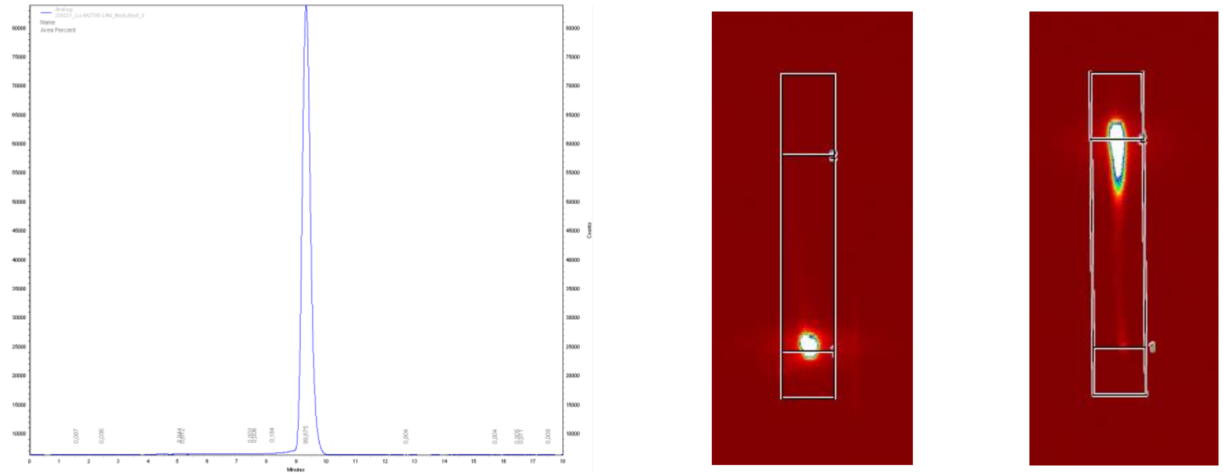

(**a**) (**b**) (**c**)

Figure S4. Typical quality control results of [^{177}Lu]Lu-AAZTA^5-LM4 produced via the module, comprising (**a**) representative HPLC radiochromatogram, (**b**) TLC foot-prints (Profile 1) and (**c**) TLC (Profile 2), all consistent with a >99% radiochemical purity of the radioligand.

Metabolic Stability of [^{111}In]In-AAZTA^5-LM4 and [^{177}Lu]Lu-AAZTA^5-LM4 in Mice

As detailed in the main manuscript, [^{111}In]In-AAZTA^5-LM4 and [^{177}Lu]Lu-AAZTA^5-LM4 were injected in 3 healthy mice each and 5 min pi blood was collected from the heart; samples were properly processed and analyzed by HPLC analysis (system 2) to detect radiometabolites potentially forming in vivo. Representative radiochromatograms of the analysis are shown in Figure S5 for [^{111}In]In-AAZTA^5-LM4 (a) and [^{177}Lu]Lu-AAZTA^5-LM4 (b).

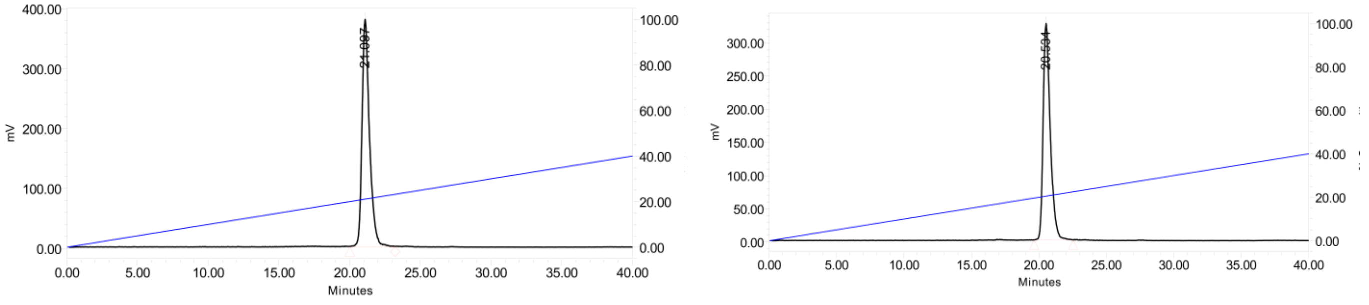

(**a**) (**b**)

Figure S5. Gamma trace of HPLC analysis of blood samples collected 5 min pi of (**a**) [^{111}In]In-AAZTA^5-LM4 (t_R =21.1 min) and (**b**) [^{177}Lu]Lu-AAZTA^5-LM4(t_R = 20.5 min) in healthy mice, showing the high metabolic stability of the radioligands in mice circulation (>98% intact); the t_R values on system 2 were determined by co-injection with a sample of the respective labeling reaction on the HPLC.

Comparative biodistribution results of In-111/Lu-177 Radioligands in Twin HEK293-SST2R/wtHEK293 Tumor-Bearing Mice

Comparative biodistribution results of [111In]In-AAZTA5-LM4 and [111In]In-DOTA-LM3 at 4 and 24 h pi in SCID mice bearing double HEK293-SST2R and "mock" wtHEK293 xenografts are included Table S3. Data is given as %IA/g and represents mean values ± sd, n= 4 per group. Experimental details and critical comments on biodistribution outcomes are discussed in the main manuscript.

Table S3. Comparative biodistribution data of [111In]In-AAZTA5-LM4 and [111In]In-DOTA-LM3 in SCID mice bearing twin HEK293-SST2R (Tu+) and wtHEK293 (Tu-) xenografts at 4 h and 24 h pi; data is expressed as %IA/g and represent average values ± sd, n = 4.

Organs/Tissues	[111In]In-AAZTA5-LM4		[111In]In-DOTA-LM3	
	4 h	24 h	4 h	24 h
Blood	0.23±0.02	0.05±0.00	0.23±0.02	0.07±0.05
Liver	1.25±0.12	0.36±0.06	2.04±0.25	0.58±0.06
Heart	0.21±0.02	0.06±0.01	0.22±0.03	0.10±0.02
Kidneys	20.08±3.99	3.34±0.77	15.36±0.58**	4.25±0.77
Stomach	2.09±0.30	0.23±0.06	0.80±0.17	0.50±0.09
Intestines	1.08 ±0.16	0.20±0.06	1.09±0.06	0.23±0.04
Spleen	0.34±0.06	0.18±0.01	0.46±0.05	0.27±0.03
Muscle	0.07±0.01	0.03±0.00	0.08±0.01	0.04±0.02
Lungs	2.63±0.39	0.31±0.02	3.14±0.48	0.59±0.12
Femur	0.46±0.04	0.13±0.01	0.62±0.02	0.32±0.07
Pancreas	6.70±0.62	0.31±0.05	9.94±0.79	1.20±0.14
Tu+	38.13±3.44	14.28±2.88	36.38±2.05	20.93±3.02****
Tu-	0.74±0.05	0.13±0.03	0.80±0.07	0.29±0.01

Statistical significant differences between the two radioligands at the same time intervals, ****, *P*<0.0001, **, *P*<0.01.

Likewise, comparative biodistribution results for [177Lu]Lu-AAZTA5-LM4 and [177Lu]Lu-DOTA-LM3 at 4, 24 and 48 h pi in the same mice model are included in Table S4.

Table S4. Comparative biodistribution data of [177Lu]Lu-AAZTA5-LM4 and [177Lu]Lu-DOTA-LM3 in SCID mice bearing twin HEK293-SST2R (Tu+) and wtHEK293 (Tu-) xenografts at 4 h, 24 h and 48 h pi; data is expressed as %IA/g and represent average values ± sd, n = 4.

Organs/Tissues	[177Lu]Lu-AAZTA5-LM4			[177Lu]Lu-DOTA-LM3		
	4 h	24 h	48 h	4 h	24 h	48 h
Blood	0.14±0.01	0.09±0.04	0.05±0.00	0.15±0.01	0.07±0.00	0.06±0.01
Liver	0.85±0.09	0.39±0.04	0.27±0.01	6.79±0.84**	3.38±0.89	1.47±0.05
Heart	0.14±0.02	0.05±0.01	0.05±0.01	0.31±0.02	0.17±0.03	0.13±0.02
Kidneys	11.55±0.34	3.85±0.85	1.45±0.13	11.52±0.26	5.14±0.92	2.47±0.67
Stomach	1.06±0.12	0.37±0.04	0.17±0.01	14.60±1.85****	8.03±1.43****	3.27±0.34
Intestines	0.77±0.08	0.24±0.04	0.08±0.00	2.71±0.18	1.52±0.21	0.78±0.09
Spleen	0.22±0.02	0.20±0.03	0.14±0.01	0.81±0.06	0.56±0.07	0.40±0.03
Muscle	0.05±0.00	0.02±0.00	0.02±0.00	0.07±0.01	0.05±0.01	0.05±0.00
Lungs	1.62±0.15	0.33±0.03	0.24±0.02	15.71±1.20****	8.88±0.26****	5.11±0.63
Femur	0.46±0.07	0.30±0.05	0.25±0.03	1.56±0.24	1.38±0.18	0.88±0.05
Pancreas	3.10±0.39	0.32±0.06	0.13±0.01	46.62±2.20****	22.96±4.32****	7.85±0.94****
Tu+	31.38±4.24	11.80±0.77	2.73±0.33	45.55±1.66****	37.57±3.42****	18.76±0.88****
Tu-	0.40±0.05	0.18±0.02	0.06±0.01	1.66±0.14	1.53±0.15	0.75±0.08

Statistical significant differences between the two radioligands at the same time intervals, ****, *P*<0.0001, **, *P*<0.01.

Radiosynthesis of [^{68}Ga]Ga-DATA^{5m}-LM4 and [^{177}Lu]Lu-AAZTA^{5}-LM4 for Patient Use

For radiolabeling of DATA^{5m}-LM4 with Ga-68, [^{68}Ga]$GaCl_3$ (200 MBq) obtained from a commercial [^{68}Ge]Ge/[^{68}Ga]Ga-generator (ITM Isotope Technologies Munich SE, Garching, Germany) was added in a vial containing a solution of DATA^{5m}-LM4 (50 µg, 32.5 nmol) in 500 µL of 1 M sodium acetate buffer pH 4.5. The reaction mixture was heated for 15 min at 95 ºC, affording [^{68}Ga]Ga-DATA^{5m}-LM4 in a radiochemical purity of >98%.

For the preparation of [^{177}Lu]Lu-AAZTA^{5}-LM4, [^{177}Lu]$LuCl_3$, obtained from the Board of Radiation and Isotope Technology, Mumbai, India (1GBq), was added into an Eppendorf Protein LoBind® centrifuge tube containing a stock solution of AAZTA^{5}-LM4 (25 nmol) in 1 M ammonium acetate buffer of pH 5.5. The mixture was heated at 30 ºC for 1 h and then passed through a Sep-Pak C-18 cartridge by EtOH/H_2O 50/50 (v/v). [^{177}Lu]Lu-AAZTA^{5}-LM4 in a radiochemical purity of >95% was then injected to the patient.

Scan and data acquisition protocol for [^{68}Ga]Ga-DATA^{5m}-LM4 PET/CT

PET/CT with [^{68}Ga]Ga-DATA^{5m}-LM4 did not require special preparation, like fasting, for the patient, but the patient was instructed to void before the radioligand injection as a prerequisite for imaging and then not to void before completion of acquisition at each time point (0.16, 1, 2, and 3 h pi). Serial whole-body PET scan studies were acquired in these time points. For acquisition, the patient was instructed to lie motionless in the supine position on the exam table. The acquisition protocol comprised an initial scout image (120 kVp, 10 mA) to define the field of view of the acquisition required, followed by a CT scan, and then PET scan was achieved at 1.5-2 min per bed. A total of four CT scans were obtained, one at each serial time point (0.16, 1, 2, and 3 h pi) before the acquisition of PET images for diagnostic as well as attenuation correction purposes. The first (at 0.16 h time point), and third (at 2 h time point) CT involved a relatively low dose CT with 120-300 mAs (range), 120 kVp, slice thickness 3.7 mm, and pitch 0.984:1. The second CT scan (at 1 h time point) was diagnostic dose CT with 120-380 mAs, 120 kVp, slice thickness 3.7 mm, and pitch 0.984:1. At this time point (1 h pi), a non-ionic, isomolar contrast medium (Iodixanol injection USP; dosage- 1 mL/kg body weight; Manus Akteeva Biopharma LLP, Ahmedabad, India) containing 320 mg I/mL was iv administered to the patient.
